# Unveiling coumarone resin as a tackifier in SBS-based PSA systems—a comprehensive viscoelasticity study

**DOI:** 10.1039/d5ra08052e

**Published:** 2025-12-09

**Authors:** Kinza Ali, Iqra Munawar, Faryal Aftab, Fatima Nawazish, Dilshad Hussain, Hind Saidani Scott, Adeela Saeed

**Affiliations:** a The Women University Multan Pakistan dradeela.chem@wum.edu.pk; b Atta Ur Rahman School of Applied Biosciences (ASAB), NUST Pakistan; c HEJ Research Institute of Chemistry, ICCBS, University of Karachi Pakistan; d University of Bristol, EEME School England UK

## Abstract

Styrene–butadiene–styrene (SBS) is an attractive block copolymer for Pressure Sensitive Adhesives (PSA) for labels, tapes, and hygiene products due to its good tack and easy processability. A key challenge in PSA formulation is overcoming the inherent trade-off between adhesion (tack) and cohesion (shear strength). This study is aimed to explore the potential of coumarone resin as a dual-phase tackifier for SBS based PSA. Owing to its aromatic structure and flexible side groups, cumarone-SBS blend offers a unique viscoelastic spectrum. Our findings confirmed strong resin–polymer compatibility and improved thermal stability. Rheological studies further showed that SBS formulations with coumarone develop a broader viscoelastic plateau and achieve a well-balanced relationship between elasticity (*G*′) and viscosity (*G*″) across frequencies relevant to PSA applications. Optimal formulation satisfies the Dahlquist criterion, achieving maximum peel strength (∼27 N/25 mm) and SAFT (∼70 °C), thus enabling tailored, cost-effective removable PSA.

## Abstract 2

Styrene–butadiene–styrene (SBS) is an attractive block copolymer for Pressure Sensitive Adhesives (PSA) for labels, tapes, and hygiene products due to its good tack and easy processability.

## Graphical abstract

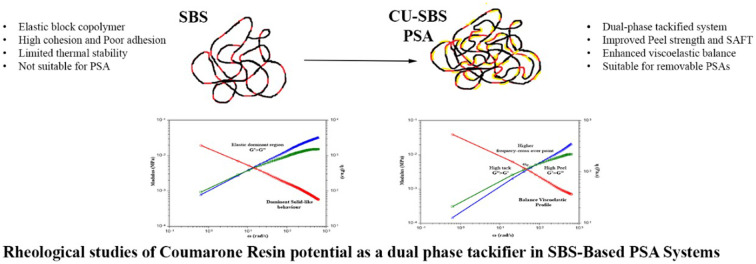

## Introduction

Pressure-sensitive adhesives (PSAs) are a unique class of viscoelastic materials capable of forming adhesive bonds to substrates with minimal pressure and without requiring curing or heat activation.^1^ These materials offer instant adhesion (tack), mechanical strength (cohesion), along with ease of application, enabling their widespread use in consumer products, packaging,^2^ hygiene products, medical applications ^3^ and industrial tapes.^4,5^ The fundamental challenge in PSA design lies in balancing the trade-off between flowability (required for surface wetting and adhesion) and mechanical robustness (essential for resisting cohesive failure under stress).

The key components of PSA typically include base polymer, tackifier, plasticizers and sometimes fillers.^6,7^ Among various base polymers, styrenic block copolymers (SBCs)-notably styrene-butadiene-styrene (SBS)—are gaining attraction for their low-cost, general-purpose applications (labels, tapes, hygiene products) owing to their good initial tack, easy processability and soft elastomeric properties.^8^ However, commercial use of SBS based PSAs is still limited due to restricted cohesive strength,^9^ inadequate thermal stability and poor long-term durability,^10^ highlighting the need for further research.^11^ To overcome these shortcomings, researchers have focused on incorporating a variety of commercially available tackifier resins in SBS for optimum performance. Tackifiers are typically low-molecular-weight resins that enhance the adhesive's ability to wet substrates (tack), maintain contact (peel adhesion), and resist deformation or failure (cohesion). The interfacial compatibility between tackifier and SBS domains significantly affects the viscoelastic behaviour and mechanical performance of the resulting adhesives. For example, tackifiers such as dicyclopentadiene resins and modified aliphatic resins show favourable interaction with the soft polybutadiene (PB) midblock, improving tack and damping.^12,13^ Conversely, aromatic resins like pentaerythritol esters are more compatible with polystyrene (PS) domains, enhancing cohesion but sometimes compromising adhesion performance.^14–16^ These findings indicate that single-phase compatibility limits performance, and that resins capable of bridging both phases may offer an optimal solution. In this regard, Coumarone resin is an attractive candidate for sustainable and economically viable PSA formulations. It is a low-cost thermoplastic resin, generally derived from C9 petroleum fractions, coal tar, or ethylene re-tar distillates *via* high-temperature polymerization. It appears as a pale yellow to dark brown brittle solid, depending on purity and polymerization degree. Its molecular structure is rich in alkyl-substituted aromatic rings, which provides a unique blend of rigidity and solubility for hydrocarbon solvents and polymer blends. It has demonstrated excellent compatibility with both natural rubber and synthetic elastomers.^17^ It is used in protective coatings, adhesives, and rubber compounding to enhance hardness, gloss, adhesion, and wear resistance.^18,19^ Its aromatic nature implies potential interaction with the PS hard blocks, while its flexible side chains may facilitate miscibility with PB segments of SBS. This dual compatibility could create a more continuous, viscoelastically balanced adhesive network, unlike conventional resins that primarily reinforce one phase.

In this study, a comprehensive viscoelastic analysis of SBS-based pressure-sensitive adhesives (PSAs) formulated with coumarone resin as a tackifier was conducted. By mapping viscoelastic properties, the balance between viscous flow and elastic resistance was analysed. These factors are directly reflected in the storage modulus (*G*′) and loss modulus (*G*″), which directly relates to tack, peel adhesion, and shear resistance. Frequency–temperature sweeps, combined with peel–tack–cohesion tests, to investigate how coumarone resin influences the mechanical and rheological profile of SBS adhesives were performed. Complementary techniques, including FTIR, TGA, DSC and SAFT were employed to assess chemical compatibility, thermal stability and morphological structures. The objective of this study is to determine whether coumarone resin can act as a dual-phase-compatible tackifier capable of enhancing both tack and cohesion, thereby contributing toward the development of cost-effective, sustainable adhesives with tailored performance for industrial and consumer applications.

## Results and discussion

### Structural and thermal characterisation

**Fig. 1:**
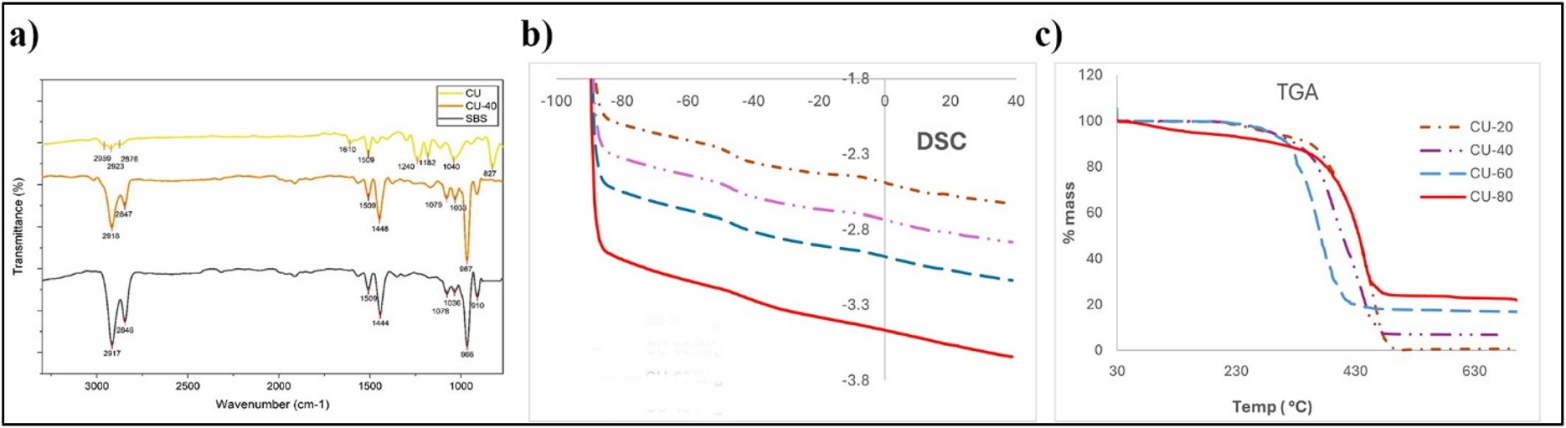
(a) FTIR spectra, showing characteristic SBS–coumarone interactions, (b) DSC thermograms, and (c) TGA curves highlighting the effect of coumarone loading on thermal stability.

The ATR-FTIR analysis of the CU-SBS blends provides clear evidence of resin incorporation and its interaction with the polymer backbone (Fig. 1a). The pure SBS spectrum is dominated by vinyl out-of-plane bending at 964 and 910 cm^−1^, aliphatic C–H stretching at 2917 and 2846 cm^−1^ and aromatic C=C stretching at 1501 cm^−1^, consistent with polybutadiene and styrene domains.^20^ Upon addition of coumarone resin (CU-40), new spectral contributions emerged, particularly the strong C–O/C–O–C stretching envelope between 1260 and 1030 cm^−1^ and enhanced aromatic absorptions at 1600–1508 cm^−1^, which overlap with the styrene-derived signals.^18^

The intensification of aliphatic C–H stretching (2920–2847 cm^−1^) further indicates the contribution of resin side chains to the overall spectral profile. More importantly, the vinyl signals from butadiene (∼964 and 910 cm^−1^) are preserved, demonstrating that the structural integrity of the polymer is maintained after blending. The coexistence and reinforcement of both resin and polymer derived peaks indicates that the presence of non-covalent π–π stacking and dipolar interactions between the aromatic rings of coumarone resin and SBS. While preserving the elastic backbone. These spectral findings suggest intermolecular associations between the resin and SBS, which are expected to influence viscoelastic balance and adhesion performance of the PSA system.

Differential scanning calorimetry (DSC) revealed that the glass transition temperature (*T*_g_) of the butadiene-rich phase shifted upward from −45 °C for CU-20 to −35 °C for CU-80 (Fig. 1b and Table 1). This rise in *T*_g_ is attributed to the aromatic structure and relatively high glass transition temperature nature of CU, whose benzofuran and indene rich aromatic rings strongly interact with polystyrene domains *via* π–π interactions while simultaneously penetrating the PS–PB interphase, likely reducing the free volume and constrain polybutadiene segmental mobility.^21,22^ Thermogravimetric analysis (TGA) supported these findings, showing a rise in degradation onset temperature from 350 °C to 400 °C (Fig. 1c and Table 1) and a DTG peak shift from 430 °C to 460 °C as resin loading increased. Moreover, the residual mass grew from 2% (CU-20) to 18% (CU-80), reflecting condensed aromatic stabilization and formation of char which caused to increase the thermal. Collectively, intermediate resin contents (CU-40 to CU-60) provided the most favourable balance between interfacial cohesion and chain mobility, optimizing tack and load bearing. In contrast, CU-80 maximized thermal stability and cohesive resistance, though at the expense of low-temperature adhesive performance.

**Table 1:** Thermal parameters of SBS–coumarone PSAs showing glass transition temperature (*T*_g_), onset/maximum temperatures (*T*_Onset_,*T*_max_) and residual mass (%) with resin concentrations

Sample ID	*T*_g_ (°C)	*T*_Onset_ (°C)	*T*_max_ (°C)	Residual mass (%)
CU-20	−45	350	432	2
CU-40	−42	370	441	8
CU-60	−38	385	455	12
CU-80	−35	400	463	18

### Viscoelastic studies

**Fig. 2:**
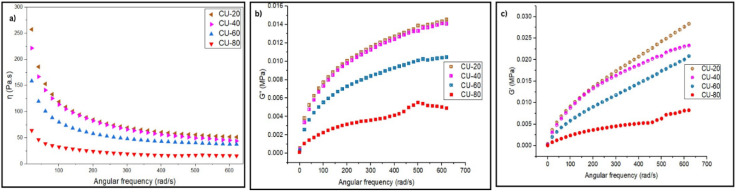
Frequency-dependent rheology of SBS–coumarone PSAs: (a) complex viscosity (*η*), (b) loss modulus (*G*″), and (c) storage modulus (*G*′) as a function of angular frequency for varying resin concentration.

All samples show a decreasing *η*′ with increasing angular frequency—classic shear-thinning behaviour (Fig. 2a).^23^ This is expected for viscoelastic PSAs: at low frequency (long timescales), chains have more time to move, so the adhesive behaves more like a high-viscosity liquid that correlates with tack/wetting capability.^24^

At high frequency, the material response more elastically, and viscosity is dropped. This high-frequency behaviour is related to ability to dissipate energy during peel. CU-20 has the highest *η*′ at low frequencies (>200 Pa·s at ∼10 rad s^−1^), meaning it's the most viscous and flowable in the long timescale regime (Table 2). This would promote good wetting. CU-40 and CU-60 show intermediate viscosities; they maintain decent viscous flow at low frequencies but are already more elastic (lower *η*′) compared to CU-20. This is often ideal for balancing tack and cohesion. CU-80 shows the lowest *η*′ across almost the whole frequency range reflecting high cohesive strength but poor low-frequency flow. The drop in *η*′ (complex viscosity) with increasing resin content is consistent with the drop in *G*″ at high resin loadings. For all samples, *G*″ increases with angular frequency, as expected for viscoelastic materials: higher frequencies give less time for relaxation, so more of the input energy is stored/dissipated per cycle (Fig. 2b). The curve slopes are similar, indicating the same basic relaxation mechanism across all resin loadings.

**Table 2:** Viscoelastic parameters of SBS–coumarone PSAs (CU-20 to CU-80) at low (6.28 rad s^−1^) and high (628 rad s^−1^) frequencies

Sample	Storage modulus, *G*′ (MPa)		Loss modulus, *G*″ (MPa)		Damping factor, tan *δ*		Viscosity, *η* (Pa s)		Crossover frequency, *ω*_c_ (rad s^−1^)
	At *ω* = 6.28 rad s^−1^	At *ω* = 628 rad s^−1^	At *ω* = 6.28 rad s^−1^	At *ω* = 628 rad s^−1^	At *ω* = 6.28 rad s^−1^	At *ω* = 628 rad s^−1^	At *ω* = 6.28 rad s^−1^	At *ω* = 628 rad s^−1^	
CU-20	5.33 × 10^−3^	2.83 × 10^−2^	2.29 × 10^−3^	1.47 × 10^−2^	1.04	0.51	686.86	51.3399	30
CU-40	1.26 × 10^−3^	2.31 × 10^−2^	1.60 × 10^−3^	1.40 × 10^−2^	1.28	0.58	612.44	43.8614	40
CU-60	1.07 × 10^−3^	2.08 × 10^−2^	1.32 × 10^−3^	1.04 × 10^−2^	1.28	0.49	338.65	37.582	80
CU-80	3.22 × 10^−4^	8.19 × 10^−3^	5.01 × 10^−4^	4.78 × 10^−3^	1.99	0.58	120.94	15.4429	75, 492

High *G*″ at low-to-moderate frequencies generally favours tack and peel because the adhesive can flow and dissipate energy during slow deformation.^25^ CU-40 is in the “sweet spot” with enough viscous flow for good wetting and high peel (more viscous dissipation → better energy loss at detachment. CU-20 has good *G*″ but lack enough cohesive strength (*G*′) to hold at elevated temperatures. CU-60 balances cohesion and dissipation but likely not as optimal for peel as CU-40. CU-80 has the lowest *η*′ and *G*″ indicating it's the least dissipative and most elastic and rigid material dominated by cohesive strength, not tack (elastic network resists shear creep).

Like *G*″, for all samples, *G*′ increases with angular frequency and follows the Dahlquist criterion to qualify as pressure-sensitive adhesives (Fig. 2c). According to the Dahlquist criterion (*G*′ < 0.3 MPa at 1 Hz, 25 °C), for pressure-sensitive adhesives. CU-20, CU-40, and CU-60 exhibit extremely low moduli (10^3^–10^4^ Pa range), ensuring effective wetting and tack.^26^ Contrary to the canonical behaviour of polymer solutions (where *G*′∝ *ω*, *G*″∝ *ω*),^27^ the storage modulus (*G*′) shows a similar trend like loss modulus (*G*″) (both moduli exhibited a notable decrease with increasing tackifier concentration) justifying the tackifier's role in plasticizing the rigid polystyrene (PS) domains, effectively diluting and softening the physical crosslinks that constitute the primary network structure.

**Fig. 3:**
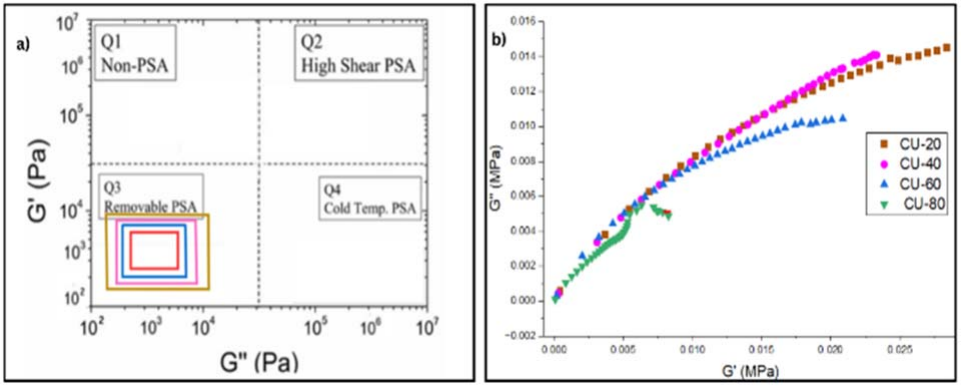
(a) Chang's viscoelastic window, (b) Cole–Cole plots, showing *G*′–*G*″ frequency response.

Chang developed the concept of the viscoelastic window (VW) as a guide to determining the application of a PSA using data obtained in the bonding frequency range (Fig. 3a).^28^ Chang's window shows that all CU formulations fall within the removable-PSA region (Q3), confirming that coumarone promotes viscous response/elastic contribution at low frequencies while maintaining sufficient elasticity at higher frequencies. This classification validates the tack-enhancing role of coumarone and explains its strong contribution to initial adhesion. Findings of Chang VW can be corelated with Cole–Cole (*G*′ *vs. G*″) plot. It provides important information related homogeneity of PSA (Fig. 3b). It provides a direct view of the relaxation spectrum of the PSA.^29^ For the coumarone-based formulations, the curve exhibits a smooth, continuous arc, indicating homogeneity in the viscoelastic response and balanced chain dynamics. This plot highlights coumarone selectively modifies the SBS matrix by interacting with both butadiene and styrene domains, shifting the viscoelastic profile toward higher damping.

Another distinct difference between low and high resin loadings is the frequency crossover point at which *G*″ = *G*′.^11^ At lower resin concentrations (CU-20, CU-40, CU-60), the storage modulus (*G*′) and loss modulus (*G*″) shows a single frequency crossover point which corresponds to the typical viscoelastic transition from viscous-dominated behaviour (*G*″ > *G*′, tan*δ* > 1) at low frequencies to elastic-dominated behaviour (*G*′ > *G*″, tan*δ* < 1) at higher frequencies. This single crossover defines the frequency window where the adhesive maintains tack (tan*δ* > 1) before transitioning into cohesive control.^27,30^ In contrast, the CU-80 formulation exhibits two distinct crossover points: the first at ∼75 rad s^−1^ and the second at ∼492 rad s^−1^. At low frequencies, *G*″ > *G*′, indicating viscous flow that facilitates substrate wetting. The first crossover signifies the onset of resin-induced stiffening, where *G*′ exceeds *G*″ and the system becomes temporarily elastic-dominated. This effect arises from excess coumarone resin saturating the polybutadiene-rich domains, restricting chain mobility and forming a pseudo-network that resists deformation. In this intermediate regime (75–492 rad s^−1^), the material behaves predominantly elastically. The second crossover (∼492 rad s^−1^) indicates the presence of multiple relaxation processes in the CU-80 blend. This dual crossover suggests that, beyond resin saturation of the soft phase, resin molecules partially partition into the polystyrene domains, creating an additional relaxation spectrum. The simultaneous contributions of polybutadiene relaxation, coumarone resin plasticisation, and styrene-domain reinforcement yield a more complex viscoelastic profile than that observed in lower-resin systems. After the second crossover, the system again becomes elastic-dominated, with *G*′ substantially greater than *G*″, reflecting the dominance of the rigid resin–styrene network.

The resin concentration significantly influences the viscoelastic transition of the SBS blends. At lower resin loading (CU-20), the material shows the longer elastic regime (*G*′ > *G*) at relatively low frequencies, with a steeper frequency dependence of the moduli. This behaviour indicates enhanced energy dissipation in the low-frequency region, which promotes effective substrate wetting and improved tack during the initial bonding stage. In contrast, higher resin concentrations sustain elevated tan*δ* values over a wider frequency range, reflecting a broader viscoelastic damping window that supports adhesion across multiple deformation rates.

The intermediate concentrations (CU-40 and CU-60) display the stable tan*δ* profiles across the frequency range, indicating an optimal balance between tackiness at low frequencies and cohesive strength at higher frequencies. CU-80 formulation, the highest tackifier concentration, shows a significant reduction in tan*δ*, suggesting a transition towards a more elastic, solid-like behaviour. CU-40 and CU-60 satisfy a good PSA criteria more effectively, displaying broad tan*δ* > 1 windows, followed by crossover into elastic dominance, consistent with their optimum peel and SAFT values.^6^ According to literature studies, effective PSAs must exhibit viscous-dominated behaviour (tan*δ* ≥ 1) at low frequencies to allow adequate substrate wetting, while transitioning to elastic-dominated behaviour (tan*δ* < 1) at higher frequencies to ensure cohesive strength during debonding.^8,11,31^ Conversely, CU-80 demonstrates two distinct crossover points, a behaviour attributed to resin-induced microphase separation.^32^

**Fig. 4:**
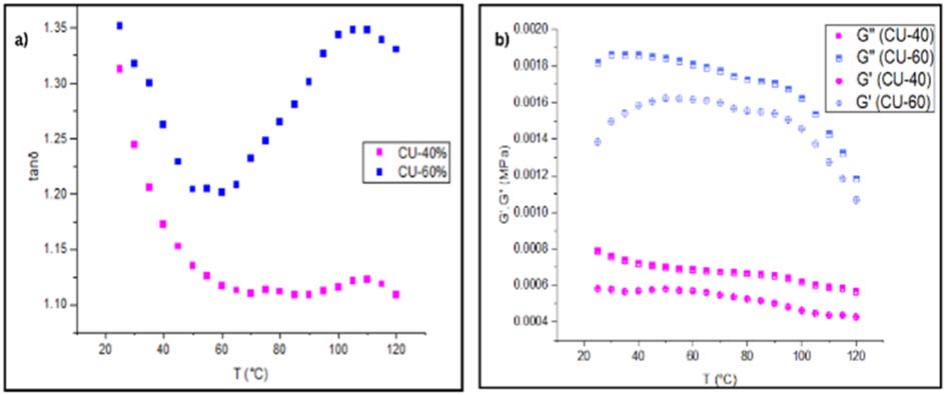
Temperature-dependent viscoelastic behaviour of SBS–coumarone PSAs, (a) tan*δ* variation with temperature for CU-40 and CU-60 (b) corresponding *G*′ and *G*″ trends.

The tan*δ*–temperature behaviour reveals that while both CU-40 and CU-60 adhesives exhibit typical viscoelastic characteristics, their high-temperature performance diverges significantly (Fig. 4a).

For CU-40, there is a sharp drop in tan*δ* from 20 °C to 60 °C, then levels off into a stable low tan*δ* plateau, indicating enhanced elastic stability. In contrast, CU-60 shows a high initial tan*δ*, which dips slightly near 60 °C and then undergoes a secondary rise above 90 °C, signifying increased viscous losses and potential morphological degradation. This ‘shape’ suggests that excessive tackifier loading compromises the adhesive's elastic backbone, leading to diminished high-temperature stability. At ≈ 100 °C, the PS domains begin to soften (*T*_g_, PS ≈ 100 °C). In CU-40, the PS physical network still holds and only a marginal viscous increase is observed. In CU-60, excessive plasticisation and softened PS cause the loss of network integrity; viscous losses dominate again. Both (CU-40, CU-60) have tan*δ* > 1 over the entire measured range, reflecting a dominant viscous response, which is favourable for tack in PSAs. CU-60 is more viscous relative to CU-40 at each temperature, indicating better wetting at elevated temperatures, but potentially more fluid under load (limited peel strength).

### Comparative performance analysis

**Fig. 5:**
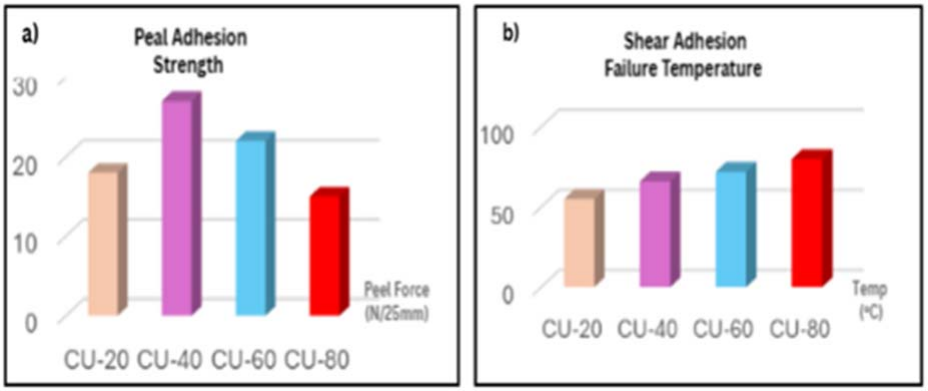
Adhesion performance of SBS–coumarone PSAs: (a) peel adhesion strength (b) shear adhesion failure temperature, SAFT (°C).

The peel strength measurements reveal a non-linear dependence on coumarone resin content. Peel adhesion is a test property of the “tack” or “aggressiveness” of an adhesive.^33^ It is maximized when the adhesive is soft and compliant, allowing it to rapidly wet out and make intimate contact with a substrate surface.^34^ At low loading (CU-20), the adhesive demonstrates moderate peel strength (∼18 N/25 mm), reflecting sufficient viscous flow of the polybutadiene mid-blocks for interfacial wetting, coupled with the cohesive reinforcement of styrene domains. When resin content is increased to CU-40, peel strength reaches a maximum (∼27 N/25 mm) (Fig. 5a).

This enhancement is attributed to the optimal resin distribution at the PS–PB interphase, where coumarone molecules enhance interfacial interactions while restricting excessive chain mobility, thereby achieving a balance between wetting and load bearing. However, further resin addition (CU-60, CU-80) results in a gradual decline in peel strength. At these higher loadings, the matrix becomes excessively constrained (anti-plasticization), reducing PB segmental flexibility and consequently limiting the adhesive's ability to dissipate energy during peeling.^35^ This trade-off reflects a common phenomenon in tackifier-modified PSAs, where intermediate resin concentrations maximize adhesion, while higher concentrations enhance cohesion at the expense of tack.^25,36^

SAFT measures the temperature at which an adhesive bond falls under a constant shear load. It is a direct indicator of the cohesive strength and thermo-mechanical stability of the adhesive itself.^37–39^ The shear adhesion failure temperature (SAFT, Fig. 5b) displays the opposite trend to peel strength, steadily increasing with resin concentration. From ∼60 °C at CU-20 to ∼90 °C at CU-80, the improvement demonstrates the positive contribution of coumarone whose compatibility with SBS restricts chain mobility and enhances thermal cohesion. At elevated loadings, the resin reduce free volume of blends, promotes condensed-phase stabilization and char formation, consistent with the TGA residual mass increase, thereby delaying cohesive failure under shear stress at high temperature. This correlation confirms that resin-rich formulations exhibit superior high-temperature resistance and cohesive integrity.

In summary, CU resin acts as a multifunctional modifier: locally constraining PB chain mobility (raising *T*_g_, enhancing thermal resistance), while globally softening the viscoelastic spectrum (reducing *G*′/*G*″). At low-to-moderate concentrations, the resin primarily plasticizes the PB-rich phase while maintaining PS-domain cohesion. This produces a viscoelastic spectrum characterized by broad tan*δ* > 1 windows and a single *G*′/*G*″ crossover, enabling strong wetting, high energy dissipation during peel, and adequate resistance to creep. At high concentration, however, the resin's role inverts; it behaves as a reinforcing filler and cause heterogeneous microdomains. The PB phase becomes saturated and immobilized, PS domains are altered, and dual relaxation processes emerge. This yields a rigid, elastic-dominated network with superior thermal and cohesive stability (high SAFT), but at the expense of flow, dissipative capacity and limited wetting, resulting in poor peel adhesion (Table 3).

**Table 3:** Peel strength and shear adhesion failure temperature (SAFT) of SBS–coumarone PSAs

Sample	Peel strength (*N*/25 mm)	Failure mode	SAFT (°C)	Failure mode
CU-20	18	Adhesive	55	Cohesive
CU-40	27	Cohesive	66	Cohesive
CU-60	22	Cohesive	72	Cohesive
CU-80	15	Adhesive	80	Cohesive

Between these extremes lies an optimal concentration window (CU-40, CU-60), where the resin enhances tack without severely compromising cohesion. In this regime, the viscoelastic profile remains responsive across a wide range of frequencies, meeting the Dahlquist criterion for bonding, sustaining dissipation during peel, and transitioning smoothly to elastic dominance under load (*G*′ > *G*″ at high *ω*). Such formulations provide the best compromise between peel strength and thermal/creep resistance, making them the suitable candidates for removable PSA applications. Taken together, the findings highlight a clear tack–cohesion trade-off governed by resin content.

Although this study demonstrates the chemical compatibility of coumarone resin with SBS, along with improved thermal stability and optimized viscoelastic characteristics for PSA applications, further research is warranted to develop complete formulations. Incorporating additional components such as oils, fillers and functional additives, will be essential to design adhesives tailored for targeted and multipurpose applications, thereby bridging laboratory findings with industrial performance requirements.

## Experimental

### Materials

The triblock copolymer used in this study is Kraton D1102, a commercial-grade styrene–butadiene–styrene (SBS) copolymer supplied by Shell Chemical Company. It possesses a molecular configuration of 10 000 g per mol polystyrene (PS) blocks at each end and a 50 000 g per mol polybutadiene (PB) midblock, resulting in a polydispersity index of 1.145. The tackifier resin employed is coumarone resin, a benzofuran-based thermoplastic compound, monomeric molecular formula C_8_H_6_O, and a molecular weight of 118.13 g per mol from Hangzhou Tiankai Enterprise Co., Ltd, China. Toluene (CAS number 108-88-3, ≥99.5% purity) is obtained from Sigma-Aldrich and used as the solvent throughout the formulation process without further purification. All chemicals are used as received.

### Sample preparation

Adhesive formulations are prepared by dissolving 1-part SBS polymer in 4-parts toluene under continuous mechanical stirring (300 rpm) for 1 hour at ambient temperature until the clear and homogenous solution was obtained. The solution is left to equilibrate under controlled conditions for 24 hours to ensure complete polymer dissolution and chain relaxation. Following this conditioning step, coumarone resin is incorporated into the SBS solution under continuous stirring (400 rpm) at different relative loadings corresponding to 0.2, 0.4, 0.6, and 0.8 parts per part of SBS corresponding to 20, 40, 60, and 80 phr (parts per hundred rubber), respectively. A control formulation without tackifier is also included for comparison.

All formulations are homogenized by manual stirring with a glass rod and subsequently conditioned for an additional 24 hours at room temperature prior to characterization and performance testing.

### Characterisation techniques

The chemical interactions between the SBS matrix and coumarone resin are investigated by Fourier-transform infrared spectroscopy in attenuated total reflectance mode (FTIR-ATR). Measurements are carried out on a PerkinElmer Spectrum 100 spectrometer over the spectral range of 4500–650 cm^−1^ with a resolution of 1 cm^−1^. Each spectrum represented the average of 20 individual scans to improve signal-to-noise ratio.^40^

Rheological properties of the formulated adhesives are evaluated using frequency and temperature sweep. First, dynamic frequency sweep tests are carried out using a Discovery HR-1 Hybrid Rheometer (TA Instruments) equipped with 25 mm parallel plates and a gap setting of approximately 1 mm.^30^ The strain amplitude is fixed at 0.1%, ensuring operation within the linear viscoelastic region (LVR).^41^ The angular frequency ranged from 628 to 0.628 rad s^−1^ with logarithmic spacing (10 points per decade). All measurements are conducted at ambient temperature (25 ± 1 °C). This setup allowed determination of key parameters such as storage modulus (*G*′), loss modulus (*G*″), complex viscosity (η*), and damping behaviour (tan*δ*).

Temperature-dependent rheological behaviour is assessed using a Discovery HR-2 Rheometer (TA Instruments) fitted with 25 mm parallel plates. In this case, the angular frequency is held constant at 6.28 rad s^−1^ with a strain amplitude of 0.1%. Measurements are carried out as the temperature increased from 25 °C to 120 °C at a controlled heating rate of 5 °C min^−1^. The resulting temperature–modulus profiles provided insight into the thermal softening and stability of the adhesive networks. The relative error in these rheological measurements did not exceed 5%.^30^

Thermal degradation characteristics are studied *via* Netzsch simultaneous thermal analyzer (STA). 5 mg of each adhesive sample is tested, using standard aluminium pan in a nitrogen atmosphere to prevent oxidative degradation. The heating rate is set at 10 °C min^−1^, and measurements are recorded from ambient temperature up to 900 °C. Differential scanning calorimetry (DSC) is performed on a TA Instruments DSC 2500. Samples ranging from 5–8 mg are hermetically sealed in standard aluminum pans and heated from −80 °C to 30 °C at a rate of 10 °C min^−1^ under a nitrogen atmosphere. This analysis allowed determination of glass transition temperature and other thermal transitions relevant to polymer phase behaviour.^42^

To assess the adhesive performance, 180° peel strength tests are conducted in accordance with ASTM D903, using a Shimadzu universal testing machine with a 10 kN load cell. Adhesive films are applied to stainless steel substrates previously cleaned with acetone. A 2 kg rubber roller is passed twice over each bonded assembly, which is then left to cure at room temperature for at least 24 hours. Adhesive bond area: 25 mm × 100 mm. Peel tests are performed at a rate of 300 mm min^−1^, and the average debonding force is recorded as peel strength.^43^

Additionally, shear adhesion failure temperature tests (SAFT) are conducted according to ASTM D3654. Each PSA sample is bonded to a stainless-steel substrate using the same rolling procedure (bonded area = 25 mm × 25 mm). A constant 1 kg shear load is applied, and the temperature is raised at a rate of 0.4 °C min^−1^, until adhesive failure occurred. The SAFT value is recorded as the temperature at which cohesive bond failure is observed.^44^

## Conclusions

The synergy between thermal, rheological, and performance analyses provides a predictive framework for resin optimization. DSC/TGA identifies resin-induced stiffening and stability, rheology translates this into measurable relaxation processes, and mechanical tests confirm the expected adhesive trade-offs. CU-40 and CU-60 formulations emerge as optimal, as their thermal and viscoelastic characteristics align with the requirements of balanced PSA applications. By contrast, CU-20 and CU-80 represent extremes in the tack–cohesion spectrum, excelling in one property while compromising the other.

This study systematically investigates the role of coumarone (CU) resin as a multifunctional tackifier in styrene–butadiene–styrene (SBS) based pressure-sensitive adhesives (PSAs). Four resin loadings (CU-20, CU-40, CU-60, CU-80 wt%) were evaluated to elucidate their influence on thermal, viscoelastic, and mechanical performance. Differential Scanning Calorimetry (DSC) revealed a progressive increase in the glass transition temperature (*T*_g_) of the polybutadiene phase, shifting from −45 °C (CU-20) to −35 °C (CU-80), indicating anti-plasticization (reduce free volume and constrain segmental mobility) and enhanced cohesive interactions. Complementary thermogravimetric analysis (TGA) demonstrated improved thermal stability, with degradation onset rising from 350 °C (CU-20) to 400 °C (CU-80), accompanied by increased char formation, consistent with the aromatic nature of CU. Rheological frequency and temperature sweeps revealed a monotonic decrease in both storage (*G*′) and loss (*G*″) moduli with increasing CU concentration, reflecting selective plasticization of the polystyrene domains while simultaneously reinforcing the polybutadiene network. At intermediate concentrations (CU-40, CU-60), broad tan*δ* > 1 windows and favorable *G*′/*G*″ crossover frequencies indicated an optimal viscoelastic balance for adhesion. In contrast, CU-80 exhibited dual crossover points (75, 492 rad s^−1^), and elasticity-dominated behavior at high frequencies, leading to suppressed peel strength but enhanced shear adhesion failure temperature (SAFT). Mechanical testing confirmed that CU-40 and CU-60 formulations achieved the best compromise between tack, peel strength, and cohesive stability, while CU-80 prioritized shear resistance at the expense of bondability. Collectively, the findings establish that coumarone does not behave as a simple plasticizer; rather, its effect in SBS-PSAs is concentration-dependent—acting initially as a network modifier and mobility regulator, and at higher loadings as a rigid reinforcing filler. These insights provide a comprehensive framework for tailoring CU-SBS blends for targeted PSA applications requiring distinct balances of adhesion, cohesion, and thermal durability.

## Author contributions

Kinza Ali led the research, performed the experiments, analysed the data, and wrote the manuscript. Dr Adeela Saeed supervised the project, provided critical guidance throughout the study, and revised the manuscript. Iqra Munawara contributed to data collection and preliminary analysis. Faryal Aftab supported the experimental procedures and assisted in data handling. Fatima Nawazish contributed to the literature review and aided in experimental work. Dilshad Hussain provided technical insights and assisted with data interpretation. Dr Hind Saidani Scott hosted Kinza Ali during her research stay at the University of Bristol, provided access to characterization facilities, and offered technical support in experimental characterisation.

## Conflicts of interest

There are no conflicts to declare.

## Data Availability

The datasets generated and analyzed during the current study are available from the corresponding author upon reasonable request.

## References

[cit1] Everaerts A. I. and Clemens L., Adhesion Science and Engineering, 2002, pp. 465–534

[cit2] Benedek I. , in Technology of Pressure-Sensitive Adhesives and Products, CRC Press, 200810-11–10-65

[cit3] Sengsuk T., Songtipya P., Kalkornsurapranee E., Johns J., Songtipya L. (2021). Polymers.

[cit4] Mapari S., Mestry S., Mhaske S. (2021). Polym. Bull..

[cit5] Benedek I. , Pressure-Sensitive Adhesives and Applications, CRC press, 2004

[cit6] Creton C. (2003). MRS Bull..

[cit7] Wang J., Guo X., Lan D., Wang Y., Huang H., Zhang C., Wu G., Zhang S., Jia Z. (2025). Carbon.

[cit8] Deng X. (2018). J. Adhes..

[cit9] Lutz J. E. and Swiggers E., Hot melt pressure sensitive adhesive composition, US Pat., US 6121344, 2000

[cit10] Phillips J. P., Deng X., Stephen R. R., Fortenberry E. L., Todd M. L., McClusky D. M., Stevenson S., Misra R., Morgan S., Long T. E. (2007). Polymer.

[cit11] da Silva S. A., Marques C. L., Cardozo N. S. M. (2012). J. Adhes..

[cit12] Lim D. H., Do H. S., Kim H. J. (2006). J. Appl. Polym. Sci..

[cit13] Lee J. (2012). Adhes. Sealants Ind..

[cit14] Sung I. K., Kim K.-S., Chin I.-J. (1998). Polym. J..

[cit15] Kim J. K., Ryu D. Y., Lee K.-H. (2000). Polymer.

[cit16] Zheng B., Song S., Chen L., Shu H., Gao D., Lan D., Li T., Liu X., Ma Y. (2025). Chem. Eng. J..

[cit17] Lin X., Wang M., Yan K. (2023). Constr. Build. Mater..

[cit18] Feng C., Hu C., Sun Z., Zhang H., Xu Z. (2024). PLoS One.

[cit19] Li C., Wang H., Lei C., Qiu H., Jiang W., Sun X., Liu Q., Zhang Y., He W. (2025). J. Energy Storage.

[cit20] Canto L., Mantovani G., Deazevedo E., Bonagamba T. J., Hage E., Pessan L. (2006). Polym. Bull..

[cit21] Doi T., Takagi H., Shimizu N., Igarashi N., Sakurai S. (2020). ACS Appl. Polym. Mater..

[cit22] Martinez C. R., Iverson B. L. (2012). Chem. Sci..

[cit23] Bian S., Zheng Z., Liu Y., Ruan C., Pan H., Zhao X. (2019). J. Mater. Chem. B.

[cit24] Fuensanta M., Vallino-Moyano M. A., Martín-Martínez J. M. (2019). Polymers.

[cit25] Ramli H., Zainal N. F. A., Hess M., Chan C. H. (2022). Chem. Teach. Int..

[cit26] Dahlquist C. A. , in Pressure-sensitive adhesives, in Treatise on Adhesion and Adhesives, ed. R. L. Patrick, Marcel Dekker, New York, 1969, vol. 2, pp. 219–260.

[cit27] Ferry J. D. , Viscoelastic Properties of Polymers, John Wiley & Sons, 1980

[cit28] Chang E. (1991). J. Adhes..

[cit29] Harrell E., Nakajima N. (1984). J. Appl. Polym. Sci..

[cit30] Mazzeo F. A. , TA Instruments Report RH082, 2002, pp. 1–8

[cit31] Kim J. K., Kim W. H., Lee D. H. (2002). Polymer.

[cit32] Constantinou A. P., Wang L., Wang S., Georgiou T. K. (2023). Polym. Chem..

[cit33] Dana S. F., Nguyen D.-V., Kochhar J. S., Liu X.-Y., Kang L. (2013). Soft Matter.

[cit34] Poh B., Chee C. (2007). Int. J. Polym. Mater..

[cit35] Stukalin E. B., Douglas J. F., Freed K. F. (2010). J. Chem. Phys..

[cit36] Pierro E. , Afferrante L. and Carbone G., in Proc. 9th Int. Conf. Fract. Fatigue Wear (FFW 2021), Springer Nature, Ghent, Belgium, 2022, pp. 139–147.

[cit37] Athavale S. P. , Hand Book of Pressure Sensitive Adhesives and Coatings: Pressure Sensitive Adhesives Technology, Notion Press, 2018

[cit38] Crosby A. J., Shull K. R. (1999). J. Polym. Sci. B Polym. Phys..

[cit39] Arrowood A., Ansari M. A., Ciccotti M., Huang R., Liechti K. M., Sanoja G. E. (2023). Soft Matter.

[cit40] Rajawasam C. W., Dodo O. J., Weerasinghe M. S. N., Raji I. O., Wanasinghe S. V., Konkolewicz D., Watuthanthrige N. D. A. (2024). Polym. Chem..

[cit41] Ricarte R. G., Shanbhag S. (2024). Polym. Chem..

[cit42] Poh B., Giam Y., Yeong F. A. (2010). J. Adhes..

[cit43] Mbithi F., Worsley P. R. (2023). J. Mech. Behav. Biomed. Mater..

[cit44] Meleshko K., Ashirbekova A., Shepelevich V., Gordienko A., Pushkareva A., Anikeenko A. (2025). Polym. Sci., Ser. D.

